# No Historical Change in Views on Aging and Their Correlates: Emerging Evidence From Germany and the United States

**DOI:** 10.1093/geroni/igab046.1111

**Published:** 2021-12-17

**Authors:** Hans-Werner Wahl, Johanna Drewelies, Sandra Duezel, Margie Lachman, Jacqui Smith, Nilam Ram, Ulman Lindenberger, Denis Gerstorf

**Affiliations:** 1 Heidelberg University, Heidelberg, Baden-Wurttemberg, Germany; 2 Humboldt University, Berlin, Berlin, Germany; 3 Max-Planck-Institute for Human Development, Berlin, Berlin, Germany; 4 Brandeis University, Brandeis University, Massachusetts, United States; 5 University of Michigan, University of Michigan, Michigan, United States; 6 Stanford University, Stanford, California, United States; 7 Humboldt University of Berlin, Humboldt University of Berlin, Brandenburg, Germany

## Abstract

To examine historical changes in views on aging, we compared matched cohorts of older adults within two independent studies that assessed differences across a two-decade interval, the Berlin Aging Studies (BASE, 1990/93 vs. 2017/18, each n = 256, Mage = 77) and the Midlife in the United States Study (MIDUS, 1995/96 vs. 2013/14, each n = 848, Mage = 67). Consistent across four different dimensions of individuals’ subjective views on aging (age felt, age appeared, desired age, attitudes towards own aging) in the Berlin Aging Studies and corroborated with subjective age felt in the MIDUS, there was no evidence whatsoever that older adults of today have more favorable views on how they age than older adults did two decades ago. We discuss reasons for our findings, including the possibility that individual age views may have become increasingly decoupled from societal age views.

